# Synergistic active and passive remote sensing with multi-feature optimization for winter wheat identification in oasis agriculture of Arid Desert Regions

**DOI:** 10.1371/journal.pone.0354298

**Published:** 2026-08-07

**Authors:** Liangzhong Cao, Jianlei Yuan, Shihua Zhu, Zuoying Tu, Xiaojie Hou, Dongmei Zhao, Shaowen Xu, Ziwei Pei, Xi Chen

**Affiliations:** 1 School of Tourism and Geography, Jiujiang University, Jiujiang, China; 2 Xinjiang Huier Agricultural Group Co., Ltd., Changji, Xinjiang, China; 3 Key Laboratory of Land Surveying and Mapping and 3S Technology Application of Jiujiang City, Jiujiang University, Jiujiang, China; 4 Jiangsu Climate Center, Jiangsu Meteorological Bureau, Nanjing, China; 5 School of Education Science, Nanjing Normal University, Nanjing, China; 6 School of Geographical Information Science, Zhejiang University of Technology, Hangzhou, China; Sathyambama Institute of Science and Technology: Sathyabama Institute of Science and Technology (Deemed to be University), INDIA

## Abstract

Accurate mapping of winter wheat is essential for food security, agricultural management, and climate adaptation strategies. We present a multi-source, phenology-aware approach for identifying winter wheat in the arid oasis agricultural landscape of Changji City (northern Xinjiang) using Sentinel-1 C-band SAR and Sentinel-2 surface reflectance imagery acquired during key growth stages in 2023. We constructed a comprehensive six-category feature set—polarimetric, textural, spectral, vegetation indices, normalized-difference indices, and temporal-difference features—and designed eleven feature-combination schemes for comparative evaluation. A Random Forest–based forward sequential feature selection (RF-FS) procedure was used to identify the most informative predictors. From an initial 171 features, RF-FS selected 27 key variables (an 84.4% reduction), dominated by SWIR bands (B11/B12), a phenology index (NDPI), and SAR polarimetric combinations; moisture-sensitive features comprised 40.7% of the selected subset. The optimized model achieved an overall accuracy (OA) of 94.60% and a Kappa coefficient of 0.9338 (producer’s accuracy [PA] = 100%). Temporal analysis indicates that April and May contributed the most discriminative information, corresponding to rapid biomass accumulation and the jointing–heading stages. Applying the optimized classifier yielded an estimated 2023 winter wheat area of approximately 93.39 km^2^ for Changji, with a relative error of 7.79% compared to statistical records. The results validate a “multi-source synergy, phenology-driven, moisture-sensitive” framework for efficient winter wheat mapping in arid desert–oasis mosaics and provide an operational basis for crop structure monitoring and agricultural risk assessment.

## Introduction

Winter wheat is one of China’s major cereal crops and plays a crucial role in national food production; therefore, maintaining its stable production capacity is of paramount importance for national food security [1,2]. Changji City, an important agricultural base in northern Xinjiang, is located within arid desert regions, and its oasis agriculture is highly sensitive to variations in water resources. Timely and accurate information on the spatial distribution of winter wheat in Changji is therefore essential for safeguarding regional food security and supporting agricultural economic development [3].

In recent years, the persistent recession of the Tianshan snowline has reduced and destabilized regional water supply, exacerbating water scarcity in Changji’s agricultural areas. At the same time, declining snow cover has weakened the insulating effect that protects overwintering crops, thereby increasing the risk of frost damage to winter wheat and other winter crops. Traditional methods for obtaining crop area and spatial distribution information—such as field surveys—are labor- and resource-intensive and cannot provide rapid, large-scale updates [4]. By contrast, the complementary use of active and passive remote sensing can overcome the limitations of optical data caused by cloud cover, enabling efficient, accurate, and wide-area extraction of land cover features and agricultural status for decision-making under water-constrained conditions.

To improve the accuracy, efficiency, and applicability of remote sensing–based winter wheat mapping, researchers have increasingly adopted synergistic approaches. In data acquisition, combining optical and radar satellite observations has been widely used to mitigate the limitations of single-source data; studies indicate that fusing optical and radar data can improve winter wheat retrieval accuracy compared to single-source approaches [5–9]. In algorithm development, machine learning and deep learning methods have been widely applied to realize data-driven pattern recognition and prediction. Deep learning semantic segmentation models show strong performance in fragmented fields and complex terrain as well as in crop damage monitoring [10–12], while machine learning classifiers such as Random Forest and Support Vector Machine are popular for their stability and interpretability [13–15]. Methodologically, common approaches include time-series vegetation indices analysis [16–18], image semantic segmentation [19–21], and feature selection techniques [8,22–26]; the latter identify the most discriminative and effective feature subsets from a high-dimensional raw feature set to improve model efficiency and accuracy.

Nevertheless, most prior studies have focused on humid and semi-humid regions, and systematic investigations targeting oasis agriculture in arid and desert environments remain relatively scarce. Existing methods also face several common challenges: high feature dimensionality and redundancy with a lack of efficient dimensionality reduction approaches; insufficient solutions for the “different object–same spectrum” problem under arid backgrounds; and difficulty balancing model complexity with computational efficiency.

Motivated by these gaps, this paper takes Changji City (Changji Hui Autonomous Prefecture, Xinjiang Uygur Autonomous Region) as the study area and constructs multi-temporal feature sets for key winter wheat phenological stages using synergistic Sentinel-1/2 observations. We derive polarimetric, textural, spectral, vegetation indices, normalized-difference indices, and temporal-difference features, organized into eleven feature-combination schemes. We then design a forward feature selection procedure based on Random Forest to identify optimal feature subsets and to reveal how different feature-type combinations cooperate to improve winter wheat discrimination. Finally, we employ a Random Forest classifier for supervised classification to map winter wheat planting areas in Changji City. The results aim to provide scientific evidence and technical support for crop structure monitoring, precision agriculture management, and yield estimation in oasis agriculture within arid and desert regions of China.

## Materials and methods

### Study region

**Fig 1:**
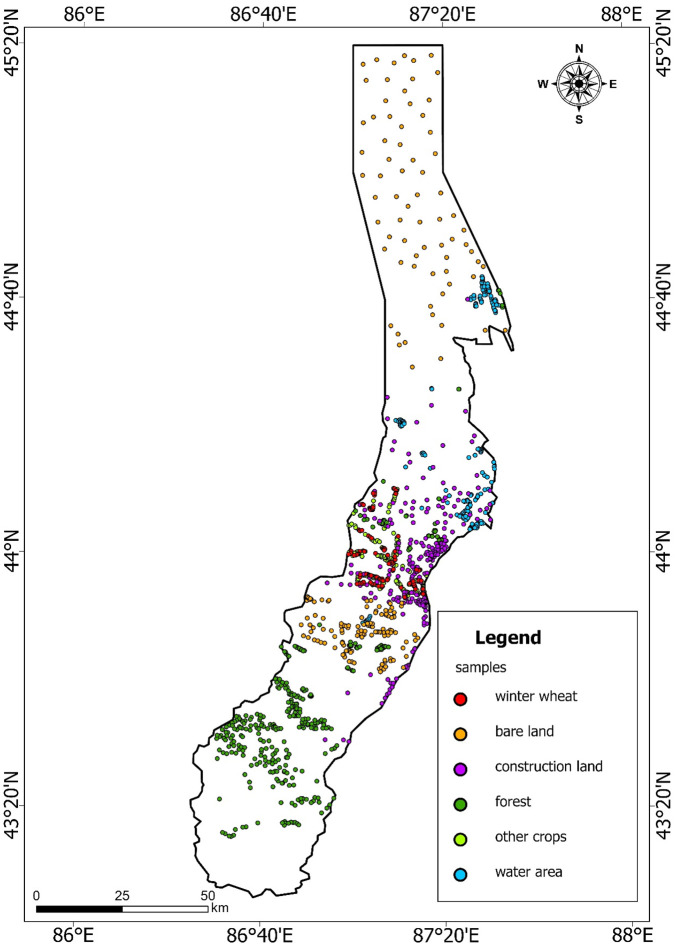
Geographical location of the study area and distribution of sampling points.

Changji City lies on the northern foothills of the Tianshan Mountains and at the southeastern edge of the Junggar Basin (Fig 1), between 43°25′–45°00′ N and 86°24′–87°37′ E, with a total area of 7,974.27 km^2^. The topography slopes from south to north: the southern region is dominated by the Tianshan mountains, the central part is a fertile alluvial plain suitable for agriculture, and the northern area is occupied by the Gurbantunggut Desert. Changji has a typical continental arid climate, with a mean annual temperature of 6.8 °C, mean annual precipitation of approximately 190 mm, and annual evaporation exceeding 1,500 mm; agricultural water supply is therefore highly dependent on meltwater from Tianshan glaciers. The accelerated retreat of the Tianshan snowline has intensified water scarcity and increased the frequency of overwinter frost damage, placing winter wheat—the region’s staple crop—under severe production risk. Winter wheat and maize are the primary grain crops in Changji; in 2023, winter wheat was planted on about 86.6 km^2^, making Changji a major grain-production base in northern Xinjiang. Accurate monitoring of cropping structure in this area is therefore of critical strategic importance for regional food security.

### Data and preprocessing

#### Remote sensing image data.

We used Sentinel-1 GRD (IW mode, VV and VH polarizations) and Sentinel-2 Level-2A surface reflectance products acquired to cover the key growth stages of winter wheat during March–May 2023. Imagery selection followed the ten-day (early/mid/late) phenological windows summarized in Table 1, which collectively capture the end of overwintering, the green-up/tillering period, and the jointing–heading stages critical for discriminating winter wheat in Changji City. Sentinel-1 provides C-band structural information (sensitive to canopy structure and moisture) with an approximate 6-day revisit, while Sentinel-2 supplies multispectral observations (10–60 m) with an effective ≈5-day revisit when S2A and S2B are combined. Sentinel-2 scenes were used in Level-2A (surface reflectance) form after atmospheric correction; cloud and snow pixels were masked using the Scene Classification Layer (SCL) together with the Normalized Difference Snow Index (NDSI).

**Table 1:** Temporal distribution of winter-wheat growth stages by ten-day periods (early/ mid/ late of each month).

Growth stage (winter wheat)	Feb			Mar			Apr			May			Jun		
	early	mid	late	early	mid	late	early	mid	late	early	mid	late	early	mid	late
Overwintering					Green-up	Resumption of vegetative growth			Stem elongation	Booting	Heading	Anthesis		Maturity

For Sentinel-1, monthly mean composites were computed for VV and VH backscatter coefficients; we additionally derived arithmetic combinations (sum, difference, product, ratio) and six GLCM texture metrics (ASM, contrast, inverse difference moment/IDM, variance, entropy, correlation) computed on the VV band. This yielded 12 radar features per month.

For Sentinel-2, we preserved 11 spectral bands (B2–B8A, B9, B11, B12) and computed 32 spectral indices (25 vegetation indices and 7 normalized-difference indices), resulting in a 43-band optical feature set per month. Features were temporally labeled with suffixes _3, _4, _5 for March, April, May respectively. In total, Sentinel-1 produced a 36-D temporal cube (12 × 3), Sentinel-2 produced a 129-D cube (43 × 3), and we additionally computed six inter-monthly phenological-change features. Lateral fusion of all features produced a 171-D feature vector per pixel.

### Sampling point data

The reliability and accuracy of sample point data play a crucial role in the precise identification of winter wheat. The sample data used in this study were collected through field surveys of agricultural crops and supplemented by visual interpretation of high-resolution historical imagery (utilizing multi-band composites) within the Google Earth Engine platform. Based on the land cover classification scheme for Changji City, Xinjiang, the sample points were categorized into the following classes: Winter Wheat, Other Crops, Forest, Built-up Land, Water Body, and Bare Land. The ‘Other Crops’ class primarily includes major cultivated species such as corn, tomatoes, cotton, and chili peppers. The number of sample points per class is as follows: Winter Wheat: 110, Other Crops: 327, Forest: 374, Built-up Land: 63, Water Body: 37, and Bare Land: 90. The spatial distribution of these samples covers the main land cover types in the study area, ensuring the representativeness of the classification model.

## Methods

**Fig 2:**
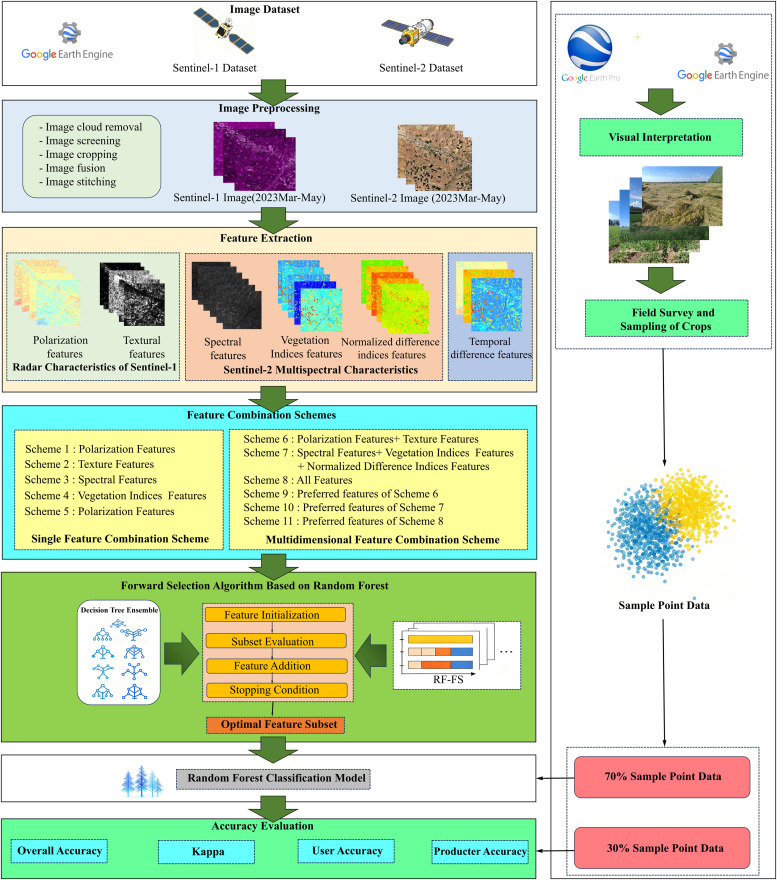
Workflow for identifying winter wheat planting areas.

The technical workflow for identifying winter wheat cultivation areas in this study is illustrated in Fig 2. The methodology comprised five main steps:

1. Data Preprocessing: Multi-temporal Sentinel imagery datasets were individually preprocessed.
2. Feature Construction: Six distinct sets of feature variables were constructed.
3. Feature Selection & Combination Evaluation: Eleven distinct feature combination schemes were designed based on the constructed feature variables. Feature optimization was then performed using a Random Forest-based Forward Feature Selection algorithm.
4. Classification & Optimal Scheme Selection: The optimized features were input into a Random Forest classification model for image classification. The accuracy of each feature combination scheme was compared to identify the optimal scheme exhibiting the highest classification performance.
5. Spatial Distribution Mapping: The optimal classification scheme was subsequently applied to generate the spatial distribution map of winter wheat across the study area.

### Feature variable

The feature variables utilized in this study were derived from active microwave remote sensing data (Sentinel-1) and multispectral remote sensing data (Sentinel-2). A total of 171 individual feature variables were constructed and categorized into six distinct types (summarized in Table 2):

**Table 2:** Feature variables.

Sensor	Feature Type	Feature Variables
**Sentinel-1**	Polarization feature	VV VH Plus=VV+VH Minus=VV-VH Multiply=VV*VH Divide=VV/VH
	Textural features	GLCM Angular Second Moment GLCM Contrast GLCM Inverse Difference Moment GLCM Variance GLCM Entropy GLCM Correlation
**Sentinel-2**	Spectral features	B2 B3 B4 B5 B6 B7 B8 B8A B9 B11 B12
	Vegetation Indices features	NDVI NDVIREP1 NDVIREP2 NDVIREP3 EVI SR DVI SAVI GNDVI TVI MTVI MCARI MCARI2 TCARI1 TCARI2 OSAVI OSAVI2 CIREP1 CIREP2 CIREP3 S2REP GLI NDre1 Ndre2 CIre
	Normalized difference indices features	NDWI NDBI NDGI NDPI NDSI NDMI NDRI
	Temporal difference features	NDVI_diff_4_3=NDVI_4-NDVI_3 NDVI_diff_5_4=NDVI_5-NDVI_4 NDVI_ratio_5_4=NDVI_4/NDVI_5 VV_diff_4_3=VV_4-VV_3 S2REP_diff_5_4= S2REP_5- S2REP_4 NDWI_diff_5_4= NDWI_5-NDWI_4

①Polarization Features:

Polarization, a fundamental property of electromagnetic waves, exhibits significant variation across different land cover types. Six polarization features were derived from Sentinel-1 data: the original VV and VH channels, which carry distinct physical scattering information, and four derived features: Plus (VV + VH), Minus (VV – VH), Multiply (VV × VH), and Divide (VV/ VH). These derived features enhance the separability of specific targets, mitigate interference from factors like terrain, and extract richer physical and biophysical information such as soil moisture and vegetation status.

②Texture Features:

Six texture features (GLCM Angular Second Moment (ASM), GLCM Contrast, GLCM Inverse Difference Moment (IDM), GLCM Variance, GLCM Entropy, and GLCM Correlation) were generated based on the Gray-Level Co-occurrence Matrix (GLCM). These features characterize crop type, planting patterns, and growth status by quantifying properties such as homogeneity, contrast, randomness, and directionality, proving particularly valuable in scenarios of spectral confusion.

③Spectral Features:

Eleven spectral band features (B2, B3, B4, B5, B6, B7, B8, B8A, B9, B11, B12) were con structed from Sentinel-2 data, covering the visible to shortwave infrared (SWIR) regions. These bands capture the reflectance characteristics of crop canopies at different wavelengths, providing the core basis for crop classification. Bands B2-B4 (visible) identify chlorophyll absorption and soil background. Bands B5-B7 (red-edge) are sensitive to crop biomass and nutrient status, enabling fine discrimination between spectrally similar crops. Bands B8 and B8A (near-infrared, NIR) quantify vegetation coverage; their differing spectral widths and positions offer complementary information. Bands B11 and B12 (SWIR) indicate crop maturity and irrigation status through their response to water content and dry matter.

④Vegetation Indices (VIs) Features:

Referencing established research [22,25–28] and considering the specific spectral characteristics of winter wheat, a set of 25 vegetation indices was constructed. This set includes the Normalized Difference Vegetation Index (NDVI), Enhanced Vegetation Index (EVI), Soil-Adjusted Vegetation Index (SAVI), among others. These indices quantify vegetation characteristics across multiple dimensions—such as chlorophyll content, water status, canopy structure, and background resistance—providing complementary spectral features for the machine learning model.

⑤Normalized Difference Indices (NDIs) Features:

Normalized Difference Indices enhance the spectral response characteristics of specific land cover types or attributes by calculating ratios between specific band combinations, thereby improving classification accuracy, interpretability, and efficiency. Based on the land cover characteristics of the Changji City study area, seven NDIs were selected as model inputs: Normalized Difference Water Index (NDWI), Normalized Difference Built-up Index (NDBI), Normalized Difference Greenness Index (NDGI), Normalized Difference Phenology Index (NDPI), Normalized Difference Snow Index (NDSI), Normalized Difference Moisture Index (NDMI), and Normalized Difference Red Edge Index (NDRI).

⑥Temporal Difference Features:

Leveraging key phenological stages of winter wheat, dynamic change features were constructed by calculating differences in multi-temporal remote sensing data. These temporal difference features capture the dynamic growth processes of crops and help mitigate interference from factors like sandy soil backgrounds and residual atmospheric noise, providing crucial time-series dimensionality for winter wheat area prediction.

### Feature combination schemes

In order to comprehensively explore the impact of active-passive remote sensing synergy on winter wheat classification accuracy, 11 groups of feature combination schemes (Table 3) were designed in this study. Scheme 1 to Scheme 5 respectively explored the performance of 5 single feature categories constructed in the model. Scheme 6 was Sentinel-1 radar information feature combination: Polarization feature and texture feature set, Scheme 7 is Sentinel-2 optical multispectral feature set, including spectral feature, vegetation index feature and normalized difference index, Scheme 8 is all constructed feature set, Schemes 9, 10 and 11 are feature optimization schemes of Schemes 6, 7 and 8 respectively.

**Table 3:** Feature combination schemes.

Scheme Number	Feature Combination Schemes	Initial feature quantity
1	Polarization Features	18
2	Texture Features	18
3	Spectral Features	33
4	Vegetation Indices Features	75
5	Normalized Difference Indices Features	21
6	Polarization Features+ Texture Features	36
7	Spectral Features+ Vegetation Indices Features+ Normalized Difference Indices Features	129
8	All Features	171
9	Preferred features of Scheme 6	36
10	Preferred features of Scheme 7	129
11	Preferred features of Scheme 8	171

### Feature selection

In this study we adopted a Random Forest–based forward feature selection frame work (RF-FS) that combines the robustness of Random Forest ensemble learning with a forward sequential (greedy) search strategy to mitigate the “curse of dimensionality” inherent in high-dimensional remote-sensing data [29]. Initially, feature importance scores for all candidate variables were computed using the explain() function in Google Earth Engine (GEE) and the features were ranked in descending order of importance. Starting from an empty set, features were then added iteratively in order of importance: at each step the next most important feature was appended to the candidate subset and the classification performance of the current subset was evaluated by ten-fold cross-validation. A newly added feature was retained only if it produced a performance improvement; the best performing subset and its associated metrics were recorded. This greedy procedure continued until all features had been tested, and the final selected feature set was taken as the subset that yielded the peak accuracy on the validation assessment.

Although the Random Forest-based forward feature selection (RF-FS) method used in this study is computationally efficient and interpretable, it is inherently a greedy algorithm and may converge to locally optimal subsets rather than the global optimum. To mitigate this risk, we initialized the selection process with a ranked importance list derived from the full feature set using GEE’s explain() function, which provides a stable starting point. Moreover, Random Forest’s inherent randomness and ensemble structure help reduce the variance in feature importance estimates. Nevertheless, future work could incorporate more robust strategies such as recursive feature elimination (RFE) or stability selection based on multiple bootstrap runs to further enhance the generalizability and robustness of the selected feature subsets.

### Random Forest classifier

The Random Forest (RF) algorithm is an ensemble learning method built from decision trees. RF constructs multiple trees by applying bootstrap sampling to the training data to form diverse training subsets; at each node split, the algorithm searches for the optimal split from a randomly chosen subset of predictor variables. Individual tree outputs are then aggregated via majority voting (classification) or averaging (regression), enabling the ensemble to capture complex, nonlinear patterns and to achieve high predictive accuracy. RF naturally provides measures of feature importance that facilitate subsequent data preprocessing and feature selection. Owing to its random sampling and randomized feature selection mechanisms, RF reduces overfitting risk and exhibits strong robustness to high dimensionality and noisy disturbances (e.g., cloud and shadow in remotely sensed imagery), making it well suited for crop recognition tasks in complex environments [30–32].

To ensure model stability and avoid overfitting, we fixed the following hyperparameters for the Random Forest classifier: number of trees (n_estimators) = 100, minimum samples per leaf node (min_samples_leaf) = 1, bootstrap sampling enabled (each tree trained on ~63.2% of the data, the default behavior), and a fixed random seed (random_state = 0) to control stochasticity.

### Accuracy assessment

Thirty percent of the samples were randomly withheld as an independent validation set. Classification accuracy was evaluated using the confusion matrix computed by GEE’s errorMatrix() function. From the confusion matrix we derived OA, the Kappa coefficient, and per-class user’s and producer’s accuracies as the metrics for assessing the classification performance.

## Results and discussion

### Feature-importance evaluation

**Fig 3:**
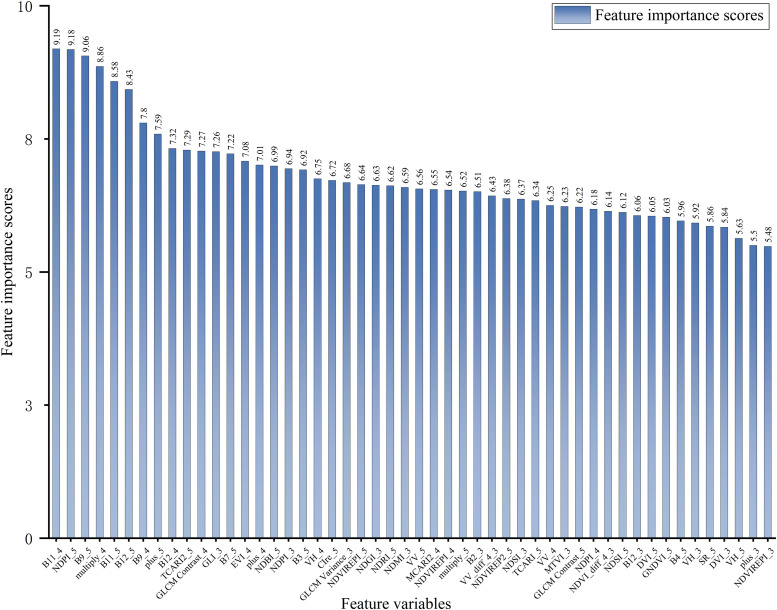
Importance score of feature variables.

A total of 171 features, derived from synergistic Sentinel-1 and Sentinel-2 active–passive remote sensing, were selected for identifying winter wheat cultivation areas in Changji. Feature importance scores for all candidate variables were computed on the Google Earth Engine (GEE) platform using the explain() function (Fig 3). Because the full feature set is large, Fig 3 presents only the top 50 features ranked by importance score.

Shortwave infrared (SWIR) bands (B11, B12) and the phenological index (NDPI) exhibited very high importance for winter wheat detection. Several SWIR-based features—e.g., B11_4, B11_5, B12_5, and B12_4—ranked within the top 10 most important predictors; these SWIR features are highly sensitive to canopy water content and dry matter, indicating that spatiotemporal mismatches in water supply and demand are a key limiting factor for winter wheat yield in this arid region. The May phenological index (NDPI_5) received an importance score of 9.18, second only to B11_4. In May, the crop canopy reaches peak closure, and because NDPI fuses red-edge and near-infrared information, it can accurately capture this phenological inflection while reducing bare-soil background interference.

Multiply_4 (the product of VV and VH) was the most important feature among non-optical features, with an importance score of 8.86. The product of VV and VH can amplify vegetation structure signals and suppress specular scattering noise from alluvial plain soils. This reflects the complementary value of active microwave remote sensing and optical remote sensing.

Seven of the top ten feature importance scores were in April, when crop biomass increases sharply and canopy structure changes dramatically, and the discriminability of each sensor to ground features was high. Among the top 20 importance features, only GLI3 and NDPI3 were from March, which is due to the low separability of snow cover and crop dormancy in March. Sentinel-2 features accounted for 68% (34 items) and Sentinel-1 features for 32% (16 items) of the features in the top 50 importance scores.

### Feature optimization analysis

**Fig 4:**
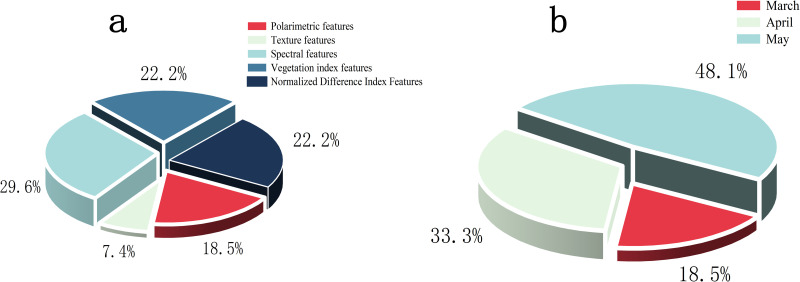
Distribution of Selected Feature Proportions: (a) By feature type; (b) By time phase.

Schemes 9–11 applied a Random Forest classifier together with a forward-selection feature-selection procedure (FS) to refine the feature sets of schemes 6–8, and then performed image classification. Scheme 11 achieved the highest classification performance: the optimized feature set contained 27 features, with an OA of 0.946 and a Kappa coefficient of 0.9338. Further analysis of the 27 selected features revealed systematic differences in their type and temporal distribution. Table 4 summarizes the counts of selected features by type and acquisition time, and Fig 4 shows their proportional distribution across types and time windows.

**Table 4:** Preferred feature distribution of Scheme 11.

Feature phase	Polarization Features	Texture Features	Spectral Features	Vegetation Indices Features	Normalized Difference Indices Features	Quantity
**Mar**		GLCM Variance_3		GLI_3	NDGI_3、 NDMI_3、 NDPI_3	5
**Apr**	VH_4、plus_4、multiply_4	GLCM Contrast _4	B9_4、B11_4、B12_4	EVI_4、MCARI2_4、		9
**May**	VV_5、plus_5		B3_5、B7_5、B9_5、B11_5、B12_5	CIre_5、NDVIREPI_5、TCARI2_5	NDPI_5、 NDRI_5、 NDPI_5	13
**Quantity**	5	2	8	6	6	27

Temporally, the 27 selected features are concentrated in late spring: May accounts for the largest share (48.1%), followed by April (33.3%), while March contributes the least (18.5%). This temporal pattern closely matches the phenological rhythm of winter wheat: May corresponds to the jointing–heading period when canopy physiological and biochemical parameters (e.g., water content and dry matter) change dramatically, producing strong remote-sensing responses and high class separability; April corresponds to the greening-to-jointing period when aboveground biomass increases rapidly and discriminative power is second only to May; March is characterized by snow cover and crop dormancy, yielding the weakest separability.

Among the radar-derived predictors retained, polarization-based features account for 71.4% of the selected radar features. Polarimetric combinations effectively suppress soil-background noise and enhance vegetation volume-scattering signals; together with optical features they form a complementary information chain that improves the identification of winter-wheat fields in desert–oasis mosaics. Shortwave-infrared (SWIR) features (B11, B12), the moisture-sensitive water-index (NDMI), and the phenological index (NDPI) are also strongly represented: 11 of the 27 selected features (40.7%) are sensitive to canopy water content, underscoring the dependence of crop discrimination in arid regions on water-stress responses. Notably, temporal-difference features were not selected, suggesting that when a sufficiently rich feature pool is available, static seasonal features may offer more robust discriminative power.

Dimensionality was reduced from the original 171 features to 27 features (an 84.4% reduction), while model accuracy improved: OA increased from 0.9353 to 0.946. These results demonstrate that the Random Forest–based forward-selection algorithm can effectively remove redundant predictors. The selected 27-dimensional feature set—dominated by SWIR optical variables, enhanced by polarization-combination radar features, and supported by NDPI throughout the phenological cycle—validates a “multi-source synergy – phenology-driven – water-sensitive” feature framework for high-accuracy winter-wheat mapping in arid environments.

### Comparison of accuracy across feature-combination schemes

Thirty percent of the samples were randomly withheld to evaluate the classification performance of eleven feature-combination schemes. Tables 5 and 6 summarize the per-scheme accuracy statistics for single-source schemes (Schemes 1–5) and multi-source/ feature-selection schemes (Schemes 6–11), respectively. The results systematically reveal how multi-source feature synergy and Random-Forest-based forward selection affect winter-wheat identification in a desert–oasis agricultural mosaic.

**Table 5:** Accuracy statistics of single-feature schemes from Scheme 1 to Scheme 5.

Category	Scheme 1		Scheme 2		Scheme 3		Scheme 4		Scheme 5	
	UA/%	PA/%	UA/%	PA/%	UA/%	PA/%	UA/%	PA/%	UA/%	PA/%
winter wheat	82.14	62.16	24.00	37.50	86.11	86.11	86.21	89.29	85.19	92.00
other crops	76.47	88.64	68.89	57.41	97.22	76.09	86.67	73.58	85.19	85.19
forest	77.78	63.64	51.72	53.57	95.31	89.71	94.68	89.00	92.94	86.81
construction land	47.89	61.82	37.93	37.93	76.47	91.55	77.78	76.24	79.76	78.82
water body	74.19	82.14	60.34	62.50	88.89	95.24	82.00	97.62	83.33	89.89
bare land	64.71	52.38	47.32	44.92	85.71	70.59	67.86	77.55	80.00	83.02
OA/%	69.44		49.21		87.14		82.84		84.70	
Kappa	0.62		0.36		0.84		0.79		0.81	

**Table 6:** Classification accuracy statistics of schemes 6-11 based on multi-dimensional feature collaboration.

Category	Scheme 6		Scheme 7		Scheme 8		Scheme 9		scheme 10		Scheme 11	
	UA/%	PA/%	UA/%	PA/%	UA/%	PA/%	UA/%	PA/%	UA/%	PA/%	UA/%	PA/%
winter wheat	73.91	77.27	86.49	88.89	94.12	96.97	86.96	80.00	89.19	91.67	97.06	100.00
other crops	88.64	86.67	81.82	91.84	97.62	91.11	86.36	90.48	85.45	95.92	92.86	92.86
forest	80.00	84.51	90.38	94.00	92.86	92.86	78.67	79.73	94.23	98.00	94.64	94.64
construction land	76.67	73.02	89.55	82.19	91.67	90.41	73.33	73.33	97.01	87.84	94.44	89.47
water body	89.23	81.69	100.00	94.74	89.58	97.73	90.77	88.06	100.00	98.18	91.67	100.00
bare land	66.67	87.50	100.00	100.00	100.00	96.30	71.43	75.00	100.00	94.44	100.00	96.30
OA/%	81.25		90.43		93.53		81.60		93.97		94.60	
Kappa	0.77		0.88		0.92		0.77		0.92		0.93	

Single-source schemes (Schemes 1–5) produced relatively low accuracies: OA ranged from 49.21% to 87.14%. Spectral (optical) features (Scheme 3) were the best single-source option, achieving OA = 87.14% and Kappa = 0.84, which confirms the fundamental role of optical information for crop discrimination. Texture features (Scheme 2) yielded the poorest performance (OA = 49.21%), indicating that radar texture alone is insufficient for reliable discrimination in complex land-cover settings. Under single-feature schemes the maximum UA for winter wheat reached 86.21% (Scheme 4) and the maximum PA reached 92% (Scheme 5), which highlights the limitations of any single information source for capturing the full suite of winter-wheat phenological characteristics.

Integrating multiple feature types produced substantial accuracy gains. The radar feature combination (polarization + texture; Scheme 6) achieved OA = 81.25%, an increase of about 32% relative to the single radar scheme, though winter-wheat UA (73.91%) and PA (77.27%) remained modest. The optical feature combination (spectra + vegetation indices + normalized indices; Scheme 7) reached OA = 90.43%, with winter-wheat UA and PA improving to 86.49% and 88.89%, respectively, demonstrating the benefit of complementary optical descriptors. The full active–passive feature fusion (Scheme 8) produced the best performance among the non-selected cases, with OA = 93.53% and Kappa = 0.92; winter-wheat UA and PA rose to 94.12% and 96.97%, respectively—further validating the complementary advantage of combining Sentinel-1 and Sentinel-2 derived predictors for complex oasis agriculture.

**Fig 5:**
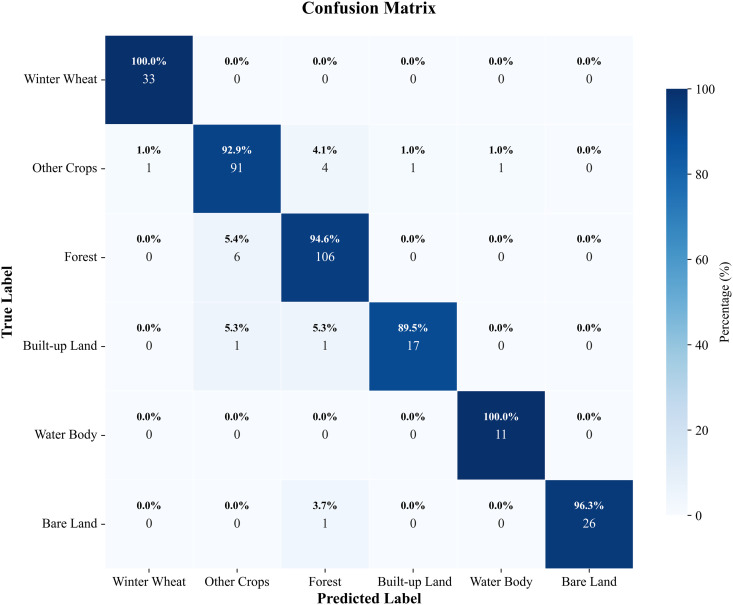
The confusion matrix for Scheme 11.

Feature optimization schemes (Schemes 9–11) significantly improved classification accuracy and optimized feature structure. In the radar feature combination, Scheme 9 optimized the 36 features of Scheme 6, and the UA of winter wheat improved from 73.91% to 86.96%. The optimization process greatly enhanced winter wheat recognition. However, the accuracy for non-agricultural land such as forest and built-up land showed a downward trend, which confirms the limitations of a single sensor in complex land scenes. After Scheme 10 optimized the 129 features of Scheme 7, OA increased from 90.43% to 93.97%. The optimization of the full feature combination realized a synergistic improvement in accuracy and efficiency. Scheme 11 selected 27 features from the 171 features of Scheme 8 by reducing dimensionality by 84.4%, and OA increased from 93.53% to 94.60%. As shown in Fig 5, the feature optimization scheme enabled the model to overcome the “curse of dimensionality” and demonstrated that only 27 “fewer but better” features are needed to surpass the full feature set for crop recognition in desert–oasis areas, achieving “dimension reduction without precision loss.”

These results demonstrate that the Random-Forest-based forward selection can effectively mitigate the “curse of dimensionality”: a compact, high-discrimination 27-dimensional feature set (dominated by SWIR optical variables, polarization-combination radar features, and phenology-sensitive indices) outperformed the full feature pool. In short, multi-source synergy combined with targeted feature reduction yields “less-but-better” feature architectures that improve classification accuracy and efficiency for winter-wheat mapping in arid oasis environments.

### Classification results

#### Comparison of classification outputs for feature-combination schemes.

**Fig 6:**
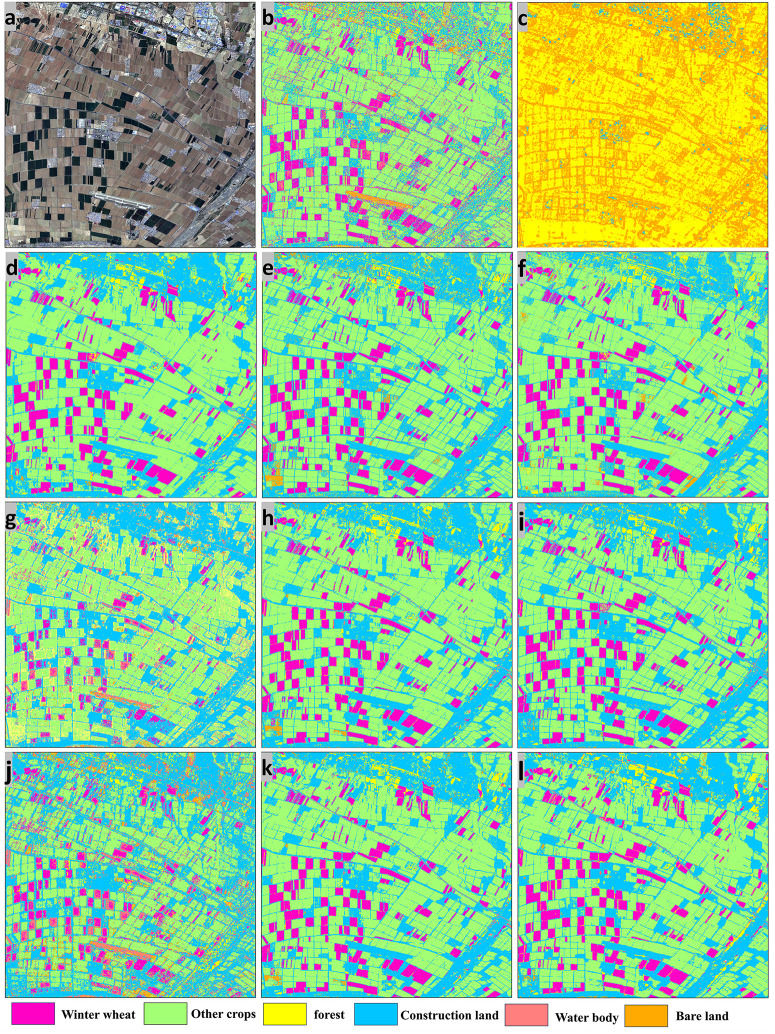
Localized Classification Results for Winter Wheat: (a) Heading stage Sentinel-2 True Color Composite; (b-l) Classification outputs of Schemes 1-11.

We selected areas within the study region characterized by dense winter-wheat plantings and well-defined field boundaries to compare classification outputs produced by each feature-combination scheme (Fig 6). The classification maps derived from radar-only feature combinations exhibit pronounced “salt-and-pepper” noise. This behavior stems from the coherent imaging mechanism of SAR: phase interference among numerous small surface scatterers produces random bright and dark speckles across otherwise homogeneous surfaces (e.g., croplands). In addition, ambiguity in polarimetric scattering between soil and vegetation, amplification of local noise by texture operators, and the pixel-wise decision rules of classifiers (which can learn noise patterns in high-dimensional feature spaces) together contribute to spotty misclassification.

Fig 6c (Scheme 2: texture features) shows the poorest performance: winter-wheat areas are largely misclassified or omitted. Texture descriptors have low sensitivity to homogeneous crop canopies; when the analysis window spans furrows or field ridges, strong contrast at soil–crop boundaries dominates the signal, causing the classifier to label ridges and non-crop structures as the primary class while treating the homogeneous winter-wheat canopy as “background noise.”.

Optical-feature combinations outperform radar-only schemes, with winter-wheat user’s accuracies (UA) exceeding 85% across those cases. However, single optical subsets still display some residual salt-and-pepper noise. From the single optical results (Fig 6d–6f): the spectral-only map (Fig 6d) shows some confusion between built-up areas and winter wheat; the vegetation- indices map (Fig 6e) presents instances where woodland is misclassified as winter wheat; and the normalized-difference indices map (Fig 6f) shows a pattern broadly similar to Fig 6e but with poorer delineation of field roads. The full optical feature fusion (Fig 6h) yields improved mapping but still produces fragmented field boundaries along ridges.

The full active–passive fusion (non-selected; Fig 6g/h as referenced) achieves better overall performance than the individual non-selected schemes, though some residual noise remains in built-up areas. Feature-selection schemes (Fig 6j–6l) consistently improve classification relative to their pre-selection counterparts: winter-wheat field boundaries become sharper, intrafield homogeneity increases, and omission/commission errors decrease. In particular, the map in Fig 6l shows markedly crisper parcel edges, enhanced intrafield uniformity, and fewer spurious misclassifications.

### Winter-wheat spatial distribution

**Fig 7:**
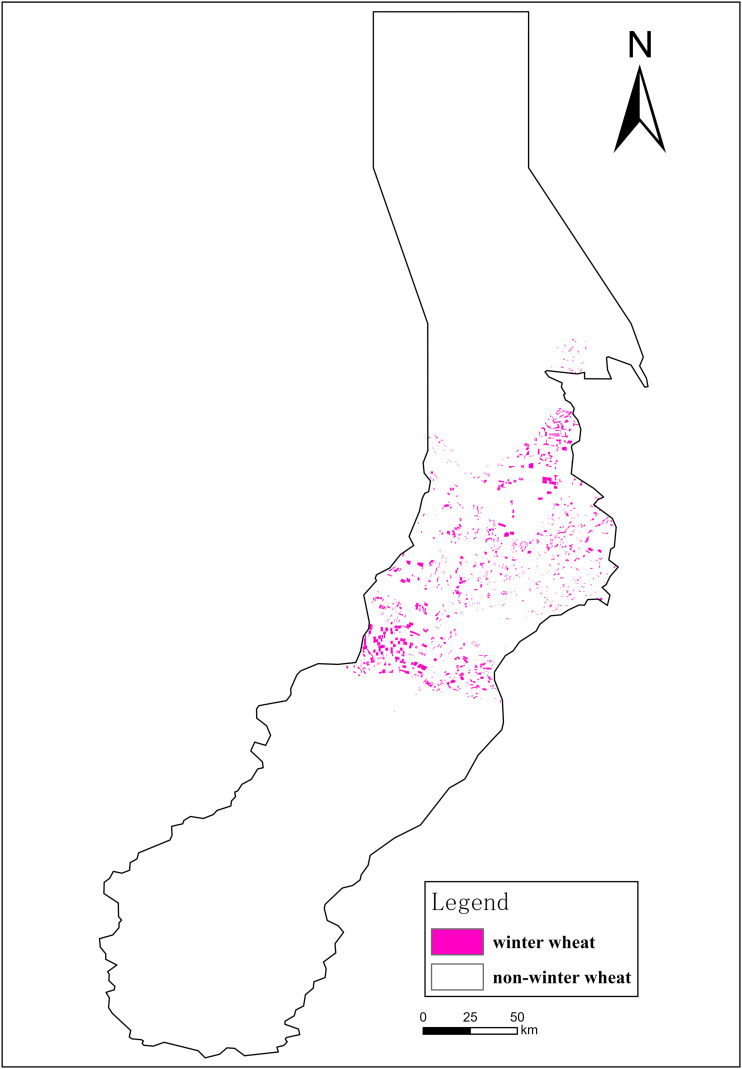
Spatial distribution of winter wheat in Changji City.

Scheme 11 — which used the 27 selected features as inputs to the Random Forest classifier — was used to map winter-wheat in Changji for 2023; the resulting spatial distribution is shown in Fig 7. Winter-wheat is concentrated in the central part of the study area, notably in Bingtuan Nonghu Farm and Bingtuan Gongqingtuan Farm, Binhu Town, Dianba Town, and Sangong Town; there are also scattered patches in the northern part of Ashili Kazakh Ethnic Township and the northern part of Liuhuangou Town. Among these areas, fields in Bingtuan Nonghu Farm and Sangong Town exhibit clear block-shaped parcels that are regularly arranged and densely distributed, whereas parcels in Yushugou Town, Erliugong Town, and parts of Binhu Town are more fragmented and sparsely distributed.

Using the RF model driven by the 27-feature set, the estimated winter-wheat area in Changji for 2023 is approximately 93.39 km^2^. For comparison, the publicly available figure in the Changji 2023 Statistical Bulletin reports a sown area of about 86.64 km^2^ [33], yielding a relative error of 7.79% [(93.39 − 86.64)/ 86.64]. The slightly larger extracted area can be attributed primarily to mixed pixels — e.g., field ridges, irrigation ditches, and narrow strips that were classified as winter-wheat — and to differences in inventory methods: official statistics typically rely on farmer self-reports or sample surveys and may omit small, scattered plots that are captured by remote sensing.

Additionally, the absence of post-classification processing, such as majority filtering or object-based smoothing, may have contributed to the inclusion of isolated spurious pixels. To evaluate the sensitivity of area estimates to such factors, we recommend future studies apply post-classification filtering and assess the impact on total area and spatial consistency. Such analysis would help disentangle genuine overestimation from differences in statistical reporting methods and improve the reliability of area estimates for operational use.

## Conclusion and discussion

This study implemented a multi-source, multi-temporal feature framework for mapping winter wheat in the study area using the Google Earth Engine (GEE) cloud platform. Feature importance scores derived from GEE were used together with a Random Forest–based forward feature selection (RF-FS) procedure to optimize the predictor set; competing feature-combination schemes were compared and the best-performing scheme was used to produce a 2023 winter-wheat map and to analyze spatial patterns. The main conclusions are:

Using RF-FS, the original 171 active–passive remote-sensing predictors were reduced to 27 key features (an 84.4% dimensionality reduction). Model performance improved after selection: OA increased from 0.9353 to 0.9460, the Kappa coefficient reached 0.9338, and PA for winter wheat attained 100%. The retained features are dominated by shortwave-infrared bands (B11, B12), a phenology index (NDPI), and a radar polarimetric combination (VV × VH). Temporally, the selected predictors are concentrated in April (33.3%) — a period of rapid biomass accumulation — and May (48.1%) — the jointing–heading phase — indicating a strong alignment between feature timing and crop phenology.

Among the eleven tested feature-combination schemes, the full active–passive feature set with RF-FS (Scheme 11) produced the highest classification accuracy, outperforming single-sensor and single-feature-type schemes. Polarimetric radar combinations effectively suppress soil-background noise and complement SWIR optical information; water-sensitive indicators (e.g., SWIR bands, NDWI) and phenology indices together account for 40.7% of the selected features, underscoring the critical role of plant water-stress responses in crop discrimination for arid oasis environments. These results validate a “multi-source synergy — phenology-driven — water-sensitive” feature framework for winter-wheat mapping in desert–oasis agricultural mosaics.

Application of Scheme 11 yielded an estimated winter-wheat area of ≈93.39 km^2^ in Changji for 2023, with major concentrations on the central alluvial plain (notably Bingtuan farms, Binhu Town, and Dianba Town); other areas (e.g., Yushugou) show more fragmented, sparse fields. Compared with the official sown area reported in the Changji 2023 Statistical Bulletin (≈86.64 km^2^), the remote-sensing estimate shows a relative difference of 7.79%. The overestimation is mainly attributable to mixed pixels (field ridges, ditches, narrow strips) being classified as cropland and to the ability of remote sensing to detect small, scattered plots that may be missed by survey-based statistics.

To reduce computational cost and focus on key phenological responses, this study limited feature extraction to three critical months (March–May). Future work should consider (a) refining temporal sampling to capture additional, specific growth stages for deeper phenology–feature analysis; (b) testing higher spatial-resolution imagery or image-fusion techniques to improve parcel delineation and reduce mixed-pixel errors; and (c) integrating more extensive ground truth and stratified sampling to further validate and generalize the proposed feature framework across broader arid agricultural regions.

While the proposed multi-source, phenology-aware feature optimization framework achieved high accuracy for winter wheat mapping in Changji City, its transferability to other arid or semi-arid regions requires further consideration due to variations in cropping systems, soil backgrounds, and environmental conditions.

First, feature selection results are inherently constrained by local phenological rhythms: the critical identification window in Changji is April and May, corresponding to rapid biomass accumulation and the jointing–heading stages, whereas in other arid regions (e.g., the Mediterranean basin, the U.S. High Plains, or Central Asia), winter wheat phenology may shift by several weeks due to variations in latitude, temperature regimes, or irrigation schedules. Therefore, prior to application, the temporal selection of remote sensing imagery must be recalibrated according to local phenology. Second, soil background variability poses a challenge: the shortwave infrared bands (B11, B12) and radar polarimetric combinations (e.g., VV × VH) selected in this study effectively suppress soil noise in alluvial plains. However, in areas with saline-alkali soils, rocky deserts, or dark clay soils, the backscatter and reflectance characteristics of land cover may change substantially, potentially affecting the separability of winter wheat from bare land or other crops. Third, the diversity of cropping patterns limits direct transferability: the “Other Crops” class in Changji mainly includes maize, tomatoes, cotton, and peppers. In regions where winter wheat is rotated with spectrally similar crops (e.g., barley or rye) or where multiple winter crops coexist, the current feature set may be insufficient to distinguish them, necessitating the introduction of additional phenological metrics or higher temporal resolution remote sensing data to avoid confusion.

To enhance the framework’s applicability across diverse arid environments, we recommend the following strategies:

①Phenology-driven temporal adjustment: Prior to application in a new region, local crop calendars should be consulted to align Sentinel-1/2 acquisitions with key growth stages (e.g., green-up, heading, senescence). A sensitivity analysis of temporal window shifts (±10 days) could help identify robust periods for discrimination.

②Soil-aware feature refinement: In regions with extreme soil types, it may be beneficial to incorporate soil-adjusted vegetation indices (e.g., SAVI, MSAVI) or polarimetric decomposition parameters (e.g., Freeman–Durden or Yamaguchi) to better isolate vegetation signals from background scattering.

③Re-optimization of feature subsets: The 27-feature set identified in this study is optimized for Changji’s conditions. When transferred, we recommend re-running the Random Forest-based forward selection (RF-FS) procedure using locally collected training samples, as feature importance rankings may shift with environmental context.

④Integration of supplementary data: In regions with complex topography or fragmented fields, very-high-resolution imagery (e.g., Planet or UAV data) or digital elevation models (DEMs) could be integrated to reduce mixed-pixel effects and improve boundary delineation.

Despite these limitations, the core logic of the framework “multi-source synergy, phenology-driven timing, and moisture-sensitive feature prioritization” is conceptually transferable. By coupling Sentinel-1/2 time series with a flexible feature selection algorithm, the approach can be adapted to support crop mapping, water resource management, and food security assessments in arid agricultural zones globally. Future work should focus on cross-regional validation and the development of region-specific feature libraries to operationalize the method at scale.

## Supporting information

S1 File Data. (ZIP)

## References

[1] Mmbando GS. Harnessing artificial intelligence and remote sensing in climate-smart agriculture: the current strategies needed for enhancing global food security. Cogent Food & Agriculture. 2025;11(1). doi: 10.1080/23311932.2025.2454354

[2] Wang Y, Zhang X, Shi J, Shen Y. [Climate change and its effect on winter wheat yield in the main winter wheat production areas of China]. Chinese J Eco-Agriculture. 2022;30(5):723–34. doi: 10.12357/cjea.20210702

[3] Jaacks L, Fraval S, Dias C. Remote sensing and food security: monitoring agriculture, ecosystems, hydrology, food environments and health outcomes. In: Space, Satellites and Sustainability. 2020;1. doi: 10.1117/12.2576496

[4] Yin J, Zhou L, Li L, Zhang Y, Huang W, Zhang H, et al. [Comparative study of multi-source remote sensing data for wheat identification and growth monitoring]. Remote Sensing Technology and Application. 2021;36(2):332–41. doi: 10.11873/j.issn.1004-0323.2021.2.0332

[5] Zhang Y, Zhou Q, Cui T, Jiang C, WU W, Zhang K, et al. Extraction of winter wheat planting area based on fused active and passive remote sensing images. In: Fifth International Conference on Computer Vision and Data Mining (ICCVDM 2024). 2024. 98. doi: 10.1117/12.3048374

[6] Zhang H, Yuan H, Du W, Lyu X. Crop Identification Based on Multi-Temporal Active and Passive Remote Sensing Images. IJGI. 2022;11(7):388. doi: 10.3390/ijgi11070388

[7] Zhang K, Cheng G, Wu W, Song X, Zhang Z, Yao S, et al. [Extraction of winter wheat planting area based on fusion of active and passive remote sensing images]. J Henan Agricultural Sciences. 2023;52(6):160–71. doi: 10.15933/j.cnki.1004-3268.2023.06.017

[8] Feng Q, Ren Y, Yao X, Niu B, Chen B, Zhao Y. [Winter wheat identification based on multi-source optical–radar data fusion in the Huang–Huai–Hai Plain]. Transactions of the Chinese Society for Agricultural Machinery. 2023;54(2):160–8. doi: 10.6041/j.issn.1000-1298.2023.02.015

[9] Ma Z, Liu C, Xue H, Li J, Fang X, Zhou J. [Winter wheat identification techniques based on fused active and passive remote sensing data in the Google Earth Engine environment]. Transactions of the Chinese Society for Agricultural Machinery. 2021;52(9):195–205. doi: 10.6041/j.issn.1000-1298.2021.09.023

[10] Sun H, Wang B, Wu Y, Yang H. Deep Learning Method Based on Spectral Characteristic Rein-Forcement for the Extraction of Winter Wheat Planting Area in Complex Agricultural Landscapes. Remote Sensing. 2023;15(5):1301. doi: 10.3390/rs15051301

[11] Yao J, Wu Y, Liu J, Wang H. Multimodal deep learning-based drought monitoring research for winter wheat during critical growth stages. PLoS One. 2024;19(5):e0300746. doi: 10.1371/journal.pone.0300746 38722916 PMC11081380

[12] Huang R, Zhao J, Yang J, Peng H, Qin X. [Spring freeze-damage identification of winter wheat in Henan based on deep learning and decadal-change simulation]. Chinese Journal of Agrometeorology. 2024;45(9):1041–52. doi: 10.3969/j.issn.1000-6362.2024.09.008

[13] Han J, Zhang Z, Cao J, Luo Y, Zhang L, Li Z, et al. Prediction of Winter Wheat Yield Based on Multi-Source Data and Machine Learning in China. Remote Sensing. 2020;12(2):236. doi: 10.3390/rs12020236

[14] Feng Z, Xiao F, Lu X, Hao B, Wang R, Zhu R. [Winter wheat classification using random forest feature selection]. Bulletin of Surveying and Mapping. 2022;(70):70–5. doi: 10.13474/j.cnki.11-2246.2022.0080

[15] Chen Y, Liu Z, Liu X, Liu T, Sun C. [Remote-sensing classification and extraction of winter wheat in Yangzhou based on machine learning algorithms]. China Agricultural Mechanization. 2024;45(8):154. doi: 10.13733/j.jcam.issn.2095-5553.2024.08.023

[16] Huang F, Xia X, Huang Y, Lv S, Chen Q, Pan Y, et al. Comparison of Winter Wheat Extraction Methods Based on Different Time Series of Vegetation Indices in the Northeastern Margin of the Qinghai–Tibet Plateau: A Case Study of Minhe, China. Remote Sensing. 2022;14(2):343. doi: 10.3390/rs14020343

[17] Yang Y, Niu Y, An W, Guo Y, Yan D. Winter wheat identification on Yunnan plateau mountains based on multi-temporal vegetation indices. Agricultural Eng. 2023;13(9):38–47. doi: 10.19998/j.cnki.2095-1795.2023.09.007

[18] Civelek N, Genç L, Akçay Ö. Predicting Winter Wheat Yield using Landsat 8-9 Based Vegetation Indices in Semi-Arid Regions. Int J Environ Geoinf. 2025;12(1):45–57. doi: 10.26650/ijegeo.1661834

[19] Zhang M, Xue Y, Zhan Y, Zhao J. Semi-supervised semantic segmentation-based remote sensing identification method for winter wheat planting area extraction. Agronomy. 2023;13(12):2868. doi: 10.3390/agronomy13122868

[20] Wang C, Zhu P, Yang S, Zhang L. A Semantic segmentation method for winter wheat in north china based on improved HRNet. Agronomy. 2024;14(11):2462. doi: 10.3390/agronomy14112462

[21] Xuqing L, Dongxue W, Yubo W, Wenbo C, Huitao G. Improved HRNet semantic segmentation model for remote sensing images of winter wheat. Trans Chin Soc Agric Eng. 2024;40(3):193–200. doi: 10.11975/j.issn.1002-6819.202308134

[22] Zhang W, Sun X, Chen Z, Ren X, Li X. Extraction of winter wheat planting area across multiple phenological periods based on feature optimization using Sentinel-1/2 data. EIES. 2025;1(01):52–72. doi: 10.63221/eies.v1i01.52-72

[23] Orynbaikyzy A, Gessner U, Mack B, Conrad C. Crop Type Classification Using Fusion of Sentinel-1 and Sentinel-2 Data: Assessing the Impact of Feature Selection, Optical Data Availability, and Parcel Sizes on the Accuracies. Remote Sensing. 2020;12(17):2779. doi: 10.3390/rs12172779

[24] Luo J, Xie M, Wu Q, Luo J, Gao Q, Shao X, et al. Early Crop Identification Study Based on Sentinel-1/2 Images with Feature Optimization Strategy. Agriculture. 2024;14(7):990. doi: 10.3390/agriculture14070990

[25] Xie Y, Wang J, Liu Y. [Recognition method for winter wheat planting areas based on Sentinel-1/2 feature selection]. Transactions of the Chinese Society for Agricultural Machinery. 2024;55(2):231–41. doi: 10.6041/j.issn.1000-1298.2024.02.022

[26] Chen J, Li H, Liu Y, Chang Z, Han W, Liu S. [Crop remote-sensing identification study based on Sentinel-2 multi-feature selection]. Remote Sensing for Natural Resources. 2023;35(3):292–300. doi: 10.6046/zrzyyg.2022272

[27] Lan S, Zhang Y, Gao T, Tong F, Tian Z, Zhang H, et al. UAV remote sensing monitoring of winter wheat tiller number based on vegetation pixel extraction and mixed-features selection. International Journal of Applied Earth Observation and Geoinformation. 2024;131:103940. doi: 10.1016/j.jag.2024.103940

[28] Zhang C, Yi Y, Wang L, Zhang X, Chen S, Su Z, et al. Estimation of the Bio-Parameters of Winter Wheat by Combining Feature Selection with Machine Learning Using Multi-Temporal Unmanned Aerial Vehicle Multispectral Images. Remote Sensing. 2024;16(3):469. doi: 10.3390/rs16030469

[29] Velthoen J, Cai J-J, Jongbloed G. Forward variable selection for random forest models. J Appl Stat. 2022;50(13):2836–56. doi: 10.1080/02664763.2022.2095362 37720244 PMC10503461

[30] Liu Y, Wang Y, Zhang J. New Machine Learning Algorithm: Random Forest. Lecture Notes in Computer Science. Springer Berlin Heidelberg. 2012. p. 246–52. doi: 10.1007/978-3-642-34062-8_32

[31] Salman HA, Kalakech A, Steiti A. Random forest algorithm overview. Babylon J Mach Learn. 2024;2024:69–79. doi: 10.58496/bjml/2024/007

[32] Rigatti SJ. Random Forest. J Insur Med. 2017;47(1):31–9. doi: 10.17849/insm-47-01-31-39.1 28836909

[33] Changji Municipal Bureau of Statistics. [Changji City 2023 National Economic and Social Development Statistical Bulletin] [Internet]. Changji: The Bureau; 2024 Jun 04 [cited 2025 Aug 27]. Available from: http://cjs.cj.gov.cn/gk/lan/931603.htm

